# Preliminary Phytochemical Screening and Antioxidant Activity of Commercial *Moringa oleifera* Food Supplements

**DOI:** 10.3390/antiox12010110

**Published:** 2023-01-02

**Authors:** Eulogio J. Llorent-Martínez, Ana I. Gordo-Moreno, María Luisa Fernández-de Córdova, Antonio Ruiz-Medina

**Affiliations:** Department of Physical and Analytical Chemistry, Faculty of Experimental Sciences, University of Jaén, Campus Las Lagunillas, E-23071 Jaén, Spain

**Keywords:** *Moringa oleifera*, food supplement, phenolics, antioxidant, HPLC-MS^n^

## Abstract

*Moringa oleifera* has been reported to possess a high number of bioactive compounds; hence, several food supplements are commercially available based on it. This work aimed to analyze the phytochemical composition and antioxidant activity of commercial food supplements. The phenolic composition of methanolic extracts was determined by using high-performance liquid chromatography with diode-array and electrospray ionization mass spectrometric detection (HPLC-DAD-ESI-MS^n^), and the antioxidant activity was assessed by ABTS^·+^ and DPPH assays. Thirty-three compounds were identified, and all the main compounds were quantified, observing that the main contribution to the phenolic profile was due to kaempferol and quercetin glucosides. The antioxidant activity in both assays agreed with the phenolic content: the higher the phenolic levels, the higher the antioxidant activity. The obtained results were compared with those previously published regarding *Moringa oleifera* leaves to establish the potential benefits of food supplement consumption in the diet.

## 1. Introduction

*Moringa* is a plant cultivated in different countries such as India, Ethiopia, the Philippines, and Sudan, and is being grown in West, East, and South Africa, tropical Asia, Latin America, the Caribbean, and the Pacific Islands. It is also known in the world as “the tree of life” because it has various parts which are used as sources of food and medicines [1]. There are 13 species of this plant, which encompass a very diverse range of growth habits or forms, from herbs and shrubs to large trees. Although they vary greatly in their form, it is very easy to distinguish a member of *Moringa* from any other plant. Large pinnate leaves characterize these species, where each leaf is divided into many leaflets. The fruits form a long and woody capsule that, when it reaches maturity, slowly opens into three valves that separate one from the other along their length, remaining attached only to the base of the fruit [2].

Of the species discussed above, *Moringa oleifera,* is the best known and most used. It is not very long-lived, about 20 years, and reaches a height of between 5–10 m. This species is native to South Asia, where it grows in the Himalayan foothills, but is widely cultivated across the tropics. Numerous studies have highlighted the advantageous influences of this plant on human health [3], which is cultivated for its edible leaves, flowers, and nutritious pods, with *M. oleifera* leaf being the most utilized part [4]. In recent years, *M. oleifera* leaves have been extensively studied due to their enormous potential as sources of functional compounds with health-promoting properties [5], especially various biological activities such as antioxidant, anti-inflammatory, anti-diabetic, anti-cancer, cardioprotective, hypocholesterolemic, hepatoprotective, antifungal, antiviral, antidepressant, and anti-asthmatic activities [6,7,8]. In addition, *M. oleifera* leaves are useful in treating neuro-dysfunctional diseases such as Alzheimer’s disease, epilepsy, and ischemic stroke [9,10]. The anti-inflammatory effects are mainly due to the large number of phenolics [11,12,13,14] present, specifically flavonoids, where numerous compounds have been described, among the most important being quercetin and kaempferol [15,16].

The phenolic composition [17] and antioxidant activity [18,19] of *M. oleifera* leaves have already been studied. Some of these works are focused on the evaluation of total phenolic content (TPC) and total flavonoid content (TFC) [20], while others also include a chromatographic study of its components [21,22,23,24,25,26,27]. In addition, various *in vitro* and *in vivo* studies have been carried out to verify the antioxidant action of the phytochemicals present in this species [28,29]. Precious-Adejoh et al. showed that *M. oleifera* extracts reduced blood glucose levels in diabetic animals and inhibit α-amylase/α-glucosidase activities, respectively [30]. Verma et al. found that the antioxidant effect of *M. oleifera* leaves on rodents was similar to that obtained with vitamin E [31].

We cannot forget that, in addition to all the properties mentioned above, *M. oleifera* is a storehouse of important nutrients. Their leaves are rich in minerals such as Ca, K, Fe, Mg, P, Zn, and Cu, and vitamins A, C, D, E, and B (B1, B2, B3, B6), and folic acid [32]. Consequently, the use of *M. oleifera* by the food industry as a natural ingredient to replace different classic preservatives and antioxidants, as well as to increase the nutritional value of certain food products, represents an interesting opportunity. To bring the properties of *M. oleifera* to consumers, a few studies have reported its incorporation into different foods (e.g., meat, biscuits, and bread). In meat products, it is used as a preservative and antioxidant additive with very good results without affecting the sensory characteristics of the final product [33,34,35]. In the field of bakery (bread, cereal gruel, and snacks such as biscuits) the objective is usually nutritional fortification [36,37]. For example, the protein and crude fiber content of wheat flour bread fortified with 5% *M. oleifera* leaves were found to increase by approximately 54% and 56%, respectively [38]. On the other hand, several studies demonstrated that a little addition of *M. oleifera* to maize flour, a major constituent of most snacks, can add nutritive value to the snack in terms of protein, energy, and minerals [39].

An alternative to enriched foods is the use of food supplements, which are defined in Directive 2002/46/EC of the European Parliament as “*food products whose purpose is to complement the normal diet and consisting of concentrated sources of nutrients or of other substances that have a nutritional or physiological effect, in simple or combined form, marketed in dosage form, that is to say capsules, pills, tablets, pills and other similar forms, powder sachets, liquid ampoules, dropper bottles and others similar forms of liquids and powders to be taken in small unit quantities*” [40]. The nutrition and health claims made on foods in their labeling are established by Regulation (EC) 1924/2006 [41], which applies, without prejudice, to the food supplement Directive 2002/46/EC [40]. Therefore, it is essential to control the composition of food supplements reported in labeling. The main objective of this work was to carry out a preliminary phytochemical screening of commercial food supplements prepared from *M. oleifera* and compare the results obtained with those previously reported for extracts of the plant. To our best knowledge, this work is the first study carried out to determine the phenolic profile and antioxidant capacity of commercially available *M. oleifera* food supplements, which can contribute significantly to the quality control of these products.

## 2. Materials and Methods

### 2.1. Sample Preparation

Six commercial food supplements containing *M. oleifera* were purchased and analyzed. The nomenclature used, the composition of each supplement, and the recommended dose by the manufacturer are summarized in Table 1.

Before performing the sample extraction, the content of 10 capsules was mixed, and 10 tablets were ground and mixed to ascertain representativity. Then, three sub-samples of each supplement were extracted and analyzed independently. Ultrasound-assisted extraction was done by placing 2.5 g of dry material in 50 mL MeOH for 10 min (Qsonica Sonicators; Newton, CT, USA) with a power of 55 W and a frequency of 20 kHz (50% power). Each sample was extracted in triplicate. Then, solutions were filtered through Whatman No.1 filters and the solvent was evaporated under reduced pressure in a rotary evaporator at 40 °C. Dried extracts (DE) were stored at −20 °C until analysis.

### 2.2. Chromatographic Analysis

The instrumentation and the chromatographic conditions are described in detail in the Supplementary Materials. Briefly, an HPLC system was connected to a DAD detector and an ion trap mass spectrometer equipped with an electrospray ionization interface, operating in negative ion mode.

MS data and analytical standards were used for compounds’ identification, whereas the quantitation was performed using UV data to construct the calibration graphs. Calibration graphs for chlorogenic acid, neochlorogenic acid, coumaric acid, quercetin, kaempferol, rutin, and vicenin-2 were prepared at concentrations 0.5–100 mg L^−1^ in MeOH. Chromatograms were recorded at 320 nm for phenolic acids and 350 nm for flavonoids. The mentioned analytical standards were used to quantify the exact compound or compounds of the same chemical family. A chromatogram showing the analytical standards used is given in Figure S1 (Supplementary Materials).

### 2.3. Antioxidant Capacity Assays

The antioxidant capacity of the selected food supplements was studied by ABTS^·+^ and DPPH assays. The results were expressed in mg Trolox equivalents per 100 g of dried extract (mg TE/g DE), mmol TE/g DE, and IC50 (50% inhibition). Details for each assay are given in Supplementary Materials.

### 2.4. Statistical Analysis

Statistical analysis was carried out using SPSS Statistics software v.22 (IBM SPSS Statistics for Windows, IBM Corp., Armonk, NY, USA). The analyses were performed in triplicate, and data are expressed as mean ± standard deviation. A one-way analysis of variance (ANOVA) with Tukey’s HSD post-hoc test (*p* < 0.05) was used to look for statistical differences among results in the quantification of compounds and antioxidant activities. Different superscripts in the corresponding tables indicate significant differences in the extracts (*p* < 0.05).

## 3. Results and Discussion

In this work, we selected food supplements containing *M. oleifera* leaves and extracts of *M. oleifera* seeds. The phenolic profile was characterized by HPLC-DAD-ESI-MS^n^, and the main compounds were quantified. Then, the antioxidant capacity was evaluated by ABTS^·+^ and DPPH assays.

### 3.1. HPLC-ESI-MS^n^ Analysis of Food Supplements’ Extracts

**Figure 1:**
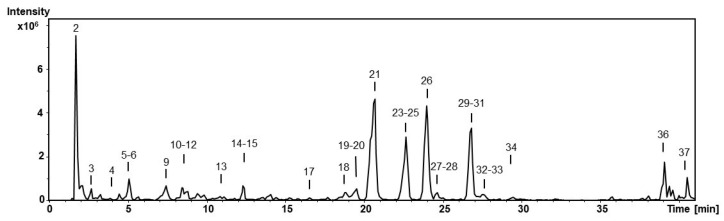
HPLC−ESI−MS^n^ base peak chromatogram of food supplement S1.

The characterization of the extracted compounds was performed by mass spectrometry, using negative ion mode (the most sensitive mode for phenolic compounds). The identification was carried out using analytical standards and data available in the scientific literature. Compounds were numbered regarding their order of elution, keeping the same numbering in all samples (Table 2). The base peak chromatogram of a food supplement is shown in Figure 1. As can be seen in Table 2, most of the characterized compounds were flavonoid glycosides, 19 out of 33 identified compounds. The phenolic profile agrees with previous reports on the composition of *M. oleifera* leaves [15,25]. Following is a brief description of the identification.

#### 3.1.1. Phenolic Acids

Compound **4** exhibited deprotonated molecular ion at *m/z* 315 and suffered the neutral loss of 162 Da to yield dihydroxybenzoic acid at *m/z* 153 (comparison with an analytical standard of protocatechuic acid), so it was characterized as its hexoside. Compounds **5** and **11** were identified as neochlorogenic acid and chlorogenic acid by comparison with analytical standards. Compound **6** exhibited the transition 179→135, typical of caffeic acid (checked with a caffeic acid analytical standard), so it was tentatively characterized as a derivative.

Compounds **9**, **12**, and **14** were identified as 3-*p*-coumaroylquinic acid, 3-feruloylquinic acid, and 4-*p*-coumaroylquinic acid, respectively, based on the hierarchical scheme proposed by Clifford et al. [42].

It is worth mentioning that although some authors mentioned gallic acid as one of the main compounds in *M. oleifera* leaves [43], we did not find this compound in any of the analyzed supplements. This is in line with the findings of other authors, who did not find gallic acid either [25].

#### 3.1.2. Flavonoids

Three apigenin *C*-glycosides were characterized: vicenin-2 (compound **15**) by comparison with an analytical standard, and vitexin (compound **18**) and isovitexin (compound **20**) based on bibliographic information [44]. The differentiation between vitexin (8-*C*-glucoside) and vitexin (6-*C*-glucoside) is due to the fragment ion at *m/z* 413, which is absent in vitexin.

Six quercetin derivatives were identified. Compound **19** was identified as rutin by comparison with an analytical standard. Compound **21** suffered the neutral loss of 162 Da (hexoside), whereas compounds **23** and **28** exhibited the neutral loss of 204 Da (acetylhexoside moiety) to yield quercetin at *m/z* 301 (fragment ions at *m/z* 179 and 151). Compound **24** was tentatively characterized as quercetin-malonyl-hexoside [45], whereas **25** was characterized as quercetin-hydroxy-methylglutaroyl-hexoside (neutral losses of 144 + 162 Da), previously reported in *M. oleifera* [46].

The same neutral losses described for quercetin glycosides were used to characterize kaempferol glycosides (compounds **22**, **26**, **29**, **30**, **31**, **32**, and **34**) and isorhamnetin glycosides (**27** and **33**).

#### 3.1.3. Other Compounds

Compound **1** was identified as citric acid by comparison with an analytical standard. Compound **2** was characterized as a disaccharide (probably diglucoside) due to the neutral loss of 162 Da (341→ 179) and the characteristic fragments of hexoside moieties (*m/z* 179, 161, 143, and 119) [47]. Compound **3** was characterized as the glucosinolate glucomoringin, previously reported in *M. oleifera* [48]. Compound **5** exhibited deprotonated molecular ion at *m/z* 315 and suffered the neutral loss of 162 Da to yield dihydroxybenzoic acid at *m/z* 153, so it was characterized as its hexoside. Compound **13** was tentatively characterized as roseoside (vomifoliolglucoside or drovomifoliol-O-β-D-glucopyranoside) based on bibliographic information [49]. Compound **35** was identified as N-feruloyltyramine [50]. This compound was only detected in food supplement S4, due to the presence of black pepper fruit, which contains this compound [51]. Hence, it was absent in all the supplements that contained only *M. oleifera*.

Compounds **36** and **37** were characterized as oxylipins oxo-dihydroxy-octadecenoic acid and trihydroxy-octadecenoic acid based on bibliographic information [52].

### 3.2. Quantification of Phytochemicals

The most abundant compounds were flavonoids, followed by phenolic acids. The following analytical standards were used: chlorogenic acid, coumaric acid, and neochlorogenic acid for phenolic acids; and quercetin, kaempferol, rutin, and vicenin-2 (an apigenin glucoside) for flavonoids. The results are shown in Table 3.

Food supplements S1, S2, and S5 presented more than 10 mg g^−1^ DE of total individual phenolic content (the sum of all the phenolics quantified by HPLC), with S5 presenting the highest amount of phenolics. However, the other supplements presented a lower concentration of phenolics, with S3 presenting the lowest concentration. Although all of them are made from *M. oleifera leaves* (except S4), these differences make it clear that the preparation of food supplements is different, as these contents of phenolics are not supposed to be based only on the origin of *M. oleifera* species. However, in all of them, the profile is similar: more than 85% of the phenolics are flavonoids (again, except in S3, with only 73% of phenolics). Among flavonoids, the main compounds are kaempferol and quercetin glycosides, in agreement with the results reported in *M. oleifera* leaves by other authors [15,25,53].

Sultana et al. [53] reported a total amount of flavonoids of 6.13 mg mg^−1^, similar to our results (2.4–12.5 mg g^−1^ DE). These same authors reported concentrations of quercetin and kaempferol of 0.281 and 0.0402 mg g^−1^, respectively, whereas we found levels of 1.4–4.7 mg g^−1^ DE for quercetin (sum of all glycosides) and 0.56–8.2 mg g^−1^ DE for kaempferol (sum of all glycosides). These differences are due to the high levels of myricetin reported by Sultana et al., whereas we did not find this flavonoid in any of the analyzed extracts.

Singh et al. [43] reported concentrations of 0.08–0.5 mg g^−1^ for chlorogenic acid, 0.05–0.5 mg g^−1^ for ferulic acid, 0.07–0.2 mg g^−1^ for kaempferol and 0.03–0.8 mg g^−1^ for quercetin. Whereas the levels of chlorogenic acid and ferulic acid are similar to the ones reported in this work (Table 3), the levels found for flavonoids by these authors were much lower, due to the different extractants used (water in their work, in contrast to methanol in ours). Other authors also reported the levels of specific phenolic compounds in *M. oleifera* [54]; however, the concentrations were given in terms of fresh weight, making the comparison not straightforward. Hence, it can be observed that a comparison in terms of the main compounds can be made (quercetin and kaempferol were the main contributors to the phenolic profile), whereas comparisons of concentration are difficult to perform.

After performing the quantitation of the most abundant compounds, we also calculated the relative contribution of all compounds using the method of area normalization. Peak areas of each compound were obtained using the precursor ion, [M-H]^-^ (Extracted Ion Chromatograms). Then, the relative contribution (in percentage) of each compound was calculated and the heat map (the darker the color, the higher the abundance) was constructed (Table 4). It can be observed that these data agree with the quantification (Table 3), observing that kaempferol and quercetin glycosides represented the highest percentage of phenolic contribution to the extracts. 

### 3.3. Antioxidant Activity

**Figure 2:**
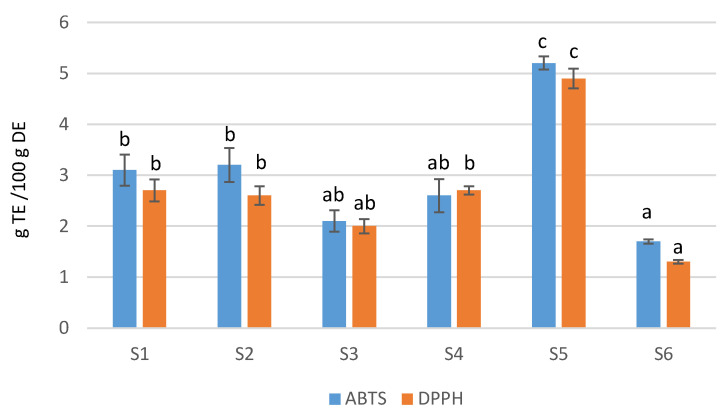
Antioxidant assays (g TE/100 g DE) for the analyzed *M. oleifera* food supplements. Means of the same assay not sharing the same letter are significantly different at *p* < 0.05 probability level.

The antioxidant capacity was evaluated utilizing the ABTS^·+^ and DPPH assays. We expressed the results in g TE (Trolox equivalents) per 100 g DE (Figure 2 and Supplementary Materials, Table S1), mmol TE/g DE (Table 5) and IC50 (amount needed to inhibit 50% of ABTS^·+^ or DPPH; Table 6). The reason to express the results of the assays in different ways is to ease comparison with other authors, as there is not consensus to express these assays in the same units.

In general, the antioxidant activity observed was in-line with the phenolic content. In this sense, supplement S5 had the highest activity, S1 and S3 presented similar capacity, and S3 and S6 had the lowest antioxidant capacity. However, there are some discrepancies; S3 and S6 presented the same antioxidant activity (no significant differences), even though S3 had less content of phenolics. This difference may be explained by the diverse antioxidant activity displayed by individual phenolics. In this case, both supplements had the same amount of quercetin-*O*-hexoside, which probably explains the similar activity. However, in general terms, the highest the phenolic content, the highest the antioxidant effect.

Braham et al. [24] reported DPPH values of 0.53 and 0.56 mmol TE/g for *M. oleifera* dried leaves, by using 70% and 50% ethanol as extraction solvents, respectively. Oldoni et al. [16] found DPPH values of 0.34 mmol TE/g of extract, obtained with 80% ethanol. Lin et al. [55] and Wu et al. [27] reported values of 0.17–0.47 and 0.07–0.15 mmol TE/g for *M. oleifera* dried leaves in the DPPH assays, respectively, with different concentrations of ethanol as the extractant and different extraction methodologies.

On the other hand, Lin et al. [55] and Wu et al. [27] reported values of 0.23–0.49 and 0.05–0.07 mmol TE/g for *M. oleifera* dried leaves in the ABTS^·+^ assays, respectively, and Oldoni et al. [16] found a value of 0.93 mmol TE/g of extract in the ABTS^·+^ assay.

When comparing our results with those previously reported by other authors, it is necessary to consider that there are differences in the solvent and the methodology used for the extraction, and in the forms of expression of results (DE in our work, in contrast to dried sample weight or extract weight in the previous works). In addition, previous studies revealed the significant influence of seasons and agroclimatic locations on the content of bioactive compounds with antiradical activity in *M. oleifera* leaves [56]. Therefore, it can be said that the results obtained in the present work in food supplements for the DPPH assay (0.05–0.20 mmol TE/g DE) and ABTS^·+^ assay (0.07–0.21 mmol TE/g DE), using methanol for extraction purposes, are of the same order as those previously reported by other authors.

In another work [57], values for IC50 of 1.02 and 1.60 mg mL^−1^ for ABTS^·+^ and DPPH assays were reported in methanol extracts of *M. oleifera* leaves. In general, these values are better than the ones found in food supplements (Table 6). However, food supplement S5 presented a similar antioxidant activity in the ABTS assay (1.26 mg mL^−1^) and slightly lower in the DPPH assay. These results agreed with the fact that S5 presented the highest phenolic concentration (Table 3).

## 4. Conclusions

In this work, we have reported the phenolic composition and antioxidant activity of six food supplements (sold in different presentations) based on *M. oleifera*, and compared the results obtained with those from other authors who analyzed *M. oleifera* fresh leaves. We found similarities in terms of phenolic profile: the main compounds were derivatives (mainly glucosides) of quercetin and kaempferol. Interestingly, we found malonyl-hexoside and acetyl-hexoside, which are not common flavonoids (the most abundant ones are usually hexoside, pentoside, deoxyhexoside, and rutinoside). However, in terms of quantitative analysis, although quercetin and kaempferol compounds were the most abundant (in agreement with previous works), the concentrations varied significantly between samples. This was an expected result, as the exact origin of *M. oleifera* plants (as well as season and agroclimatic conditions) and the preparation procedure, not provided by the different manufacturers, are probably different. Regarding the antioxidant capacity, in general, a good potential was obtained for most of the supplements; also, the results were different among them. However, as expected, there was a correlation between phenolic content and antioxidant activity: the higher the phenolic content, the higher the antioxidant activity. In our opinion, the consumption of these food supplements seems to provide a valuable source of antioxidants to the diet, although it is clear that not all the supplements provide the same amount of phenolics (which is equivalent to the antioxidant benefits).

## Figures and Tables

**Table 1:** Nomenclature used and data for each food supplement of *M. oleifera* analyzed.

Nomenclature	Presentation	Composition	Recommended Dose per Day	Recommended Dose per Day (mg *M. oleifera*)
S1	powder	*M. oleifera* leaves	5 g	5000 mg
S2	powder	*M. oleifera* leaves	3 g	3000 mg
S3	capsules	*M. oleifera* leaves (500 mg/capsule), chromium picolinate, magnesium stearate	1–2 capsules	500–1000 mg
S4	capsules	extract of *M. oleifera* seeds (300 mg/capsule) and powder of black pepper fruit, maltodextrin	1–2 capsules	300–600 mg
S5	capsules	*M. oleifera* leaves (300 mg/capsule)	2–4 capsules	600–1200 mg
S6	tablets	*M. oleifera* leaves (490 mg/tablet)	4–12 tablets	1960–5880 mg

**Table 2:** Characterization of phytochemicals found in extracts of food supplements of *M. oleifera* by HPLC-DAD-MS^n^.

No.	t*_R_* (min)	[M-H]^−^ *m/z*	m/z (% Base Peak)	Assigned Identification	S1	S2	S3	S4	S5	S6
1	1.8	191	MS^2^ [191]: 173 (10), 153 (82), 111 (100)	Citric acid *			✓	✓		
2	1.8	341	MS^2^ [341]:179 (100), 161 (81), 143 (29), 119 (45) MS^3^ [341→179]: 161 (57), 143 (65), 131 (90), 119 (100)	Disaccharide	✓	✓	✓	✓	✓	✓
3	2.6	570	MS^2^ [570]: 424 (5), 328 (26), 291 (16), 275 (18), 259 (100)	Glucomoringin	✓	✓	✓		✓	✓
4	3.9	315	MS^2^ [315]: 153 (100), 123 (8) MS^3^ [315→153]: 123 (100)	Dihydroxybenzoic acid-*O*-hexoside	✓	✓	✓		✓	✓
5	5.1	353	MS^2^ [353]: 191 (100), 179 (50), 173 (5), 135 (14)	Neochlorogenic acid *	✓	✓	✓	✓	✓	✓
6	5.1	375	MS^2^ [375]: 201 (100), 179 (52), 135 (14) MS^3^ [375→179]: 135 (100)	Caffeic acid derivative	✓	✓	✓	✓	✓	✓
7	5.8	463	MS^2^ [463]: 419 (100) MS^3^ [463→419]: 419 (100), 373 (35), 331 (45), 207 (20)	Unknown			✓			
8	6.4	628	MS^2^ [628]: 291 (100)	Unknown						
9	7.6	337	MS^2^ [337]: 163 (100) MS^3^ [337→163]: 119 (100)	3-*p*-Coumaroylquinic acid	✓	✓	✓	✓	✓	✓
10	8.4	612	MS^2^ [612]: 370 (100), 275 (75)	Unknown	✓	✓	✓		✓	✓
11	8.7	353	MS^2^ [353]: 191 (16), 179 (49), 173 (100) MS^3^ [353→173]: 155 (100), 111 (54)	Chlorogenic acid *	✓	✓	✓	✓	✓	✓
12	8.8	367	MS^2^ [367]: 193 (100) MS^3^ [367→193]: 149 (37), 134 (100)	3-Feruloylquinic acid	✓	✓	✓	✓	✓	✓
13	10.8	431	MS^2^ [431]: 385 (100), 223 (14) MS^3^ [431→385]: 223 (100), 205 (69), 161 (22), 153 (69)	Roseoside (formate adduct)	✓	✓		✓	✓	✓
14	12.1	337	MS^2^ [337]: 173 (100), 163 (7) MS^3^ [337→173]: 111 (100)	4-*p*-Coumaroylquinic acid	✓	✓	✓	✓	✓	✓
15	12.4	593	MS^2^ [593]: 575 (6), 503 (27), 473 (100), 383 (21), 353 (62)	Vicenin-2 (Apigenin 6,8-di-*C*-glucoside) *	✓	✓	✓	✓	✓	✓
16	14.4	324	MS^2^ [324]: 278 (100), 255 (53), 132 (97)	Unknown			✓	✓	✓	
17	16.6	563	MS^2^ [563]: 417 (100), 271 (27) MS^3^ [563→417]: 271 (100) MS^4^ [563→417→271]: 165 (100)	Unknown flavonoid-di-dHex	✓	✓	✓	✓	✓	✓
18	18.8	431	MS^2^ [431]: 341 (6), 311 (100) MS^3^ [431→311]: 283 (100)	Vitexin (8-*C*-glucoside-apigenin)	✓	✓	✓	✓	✓	✓
19	19.3	609	MS^2^ [609]: 301 (100) MS^3^ [609→301]: 179 (97), 151 (100)	Rutin *	✓	✓	✓	✓	✓	✓
20	19.5	431	MS^2^ [431]: 413 (6), 341 (37), 311 (100) MS^3^ [341→311]: 283 (100)	Isovitexin (6-*C*-glucoside-apigenin)	✓	✓	✓	✓	✓	✓
21	20.7	463	MS^2^ [463]: 301 (100), 179 (10), 151 (5) MS^3^ [463→301]: 179 (100), 151 (81)	Quercetin-*O*-Hex	✓	✓	✓	✓	✓	✓
22	22.5	593	MS^2^ [593]: 285 (100), 255 (10), 229 (5) MS^3^ [593→285]: 257 (100), 241 (43), 169 (35)	Kaempferol-*O*-Rut		✓		✓		✓
23	22.6	505	MS^2^ [505]: 463 (33), 301 (100), 151 (2) MS^3^ [505→301]: 271 (55), 179 (76), 151 (100)	Quercetin-*O*-acetyl-Hex	✓	✓	✓	✓	✓	✓
24	22.9	549	MS^2^ [549]: 505 (100) MS^3^ [549→505]: 463 (9), 301 (100) MS^4^ [549→505→301]: 179 (80), 151 (100)	Quercetin-malonyl-Hex	✓	✓			✓	✓
25	22.9	607	MS^2^ [607]: 463 (100), 301 (39) MS^3^ [607→463]: 301 (100), 151 (42)	Quercetin-hydroxy-methylglutaroyl-Hex	✓	✓	✓	✓	✓	
26	24.0	447	MS^2^ [447]: 285 (100), 284 (57), 255 (24) MS^3^ [447→285]: 257 (9), 255 (100)	Kaempferol-*O*-Hex	✓	✓	✓	✓	✓	✓
27	24.6	477	MS^2^ [477]: 315 (55), 314 (100) MS^3^ [477→314]: 300 (100), 271 (63)	Isorhamnetin-*O*-Hex	✓	✓	✓	✓	✓	✓
28	25.0	505	MS^2^ [505]: 463 (17), 301 (100), 151 (5) MS^3^ [505→301]: 271 (25), 255 (30), 179 (100), 151 (82)	Quercetin-*O*-acetyl hexoside	✓	✓	✓	✓	✓	✓
29	26.5	591	MS^2^ [591]: 447 (100) MS^3^ [591→447]: 285 (100)	Kaempferol-hydroxy-methylglutaroyl Hex	✓	✓	✓	✓		✓
30	26.8	533	MS^2^ [533]: 489 (100) MS^3^ [533→489]: 285 (100) MS^4^ [533→489→285]: 257 (69), 241 (60), 199 (100)	Kaempferol-malonyl-Hex	✓	✓			✓	✓
31	26.8	489	MS^2^ [489]: 285 (100) MS^3^ [489→285]: 257 (42), 255 (27), 241 (100)	Kaempferol-*O*-acetyl hexoside	✓	✓	✓	✓	✓	✓
32	27.5	489	MS^2^ [489]: 285 (100) MS^3^ [489→285]: 255 (100), 151 (77)	Kaempferol-*O*-acetyl hexoside	✓	✓			✓	✓
33	27.6	519	MS^2^ [519]: 315 (100), 300 (8) MS^3^ [519→315]: 300 (100)	Isorhamnetin-*O*-acetyl hexoside	✓	✓			✓	✓
34	29.5	489	MS^2^ [489]: 285 (100), 255 (6), 151 (4) MS^3^ [489→285]: 255 (100), 227 (30)	Kaempferol-*O*- acetyl hexoside	✓	✓	✓	✓	✓	✓
35	31.4	312	MS^2^ [312]: 178 (100), 135 (59)	N-Feruloyltyramine				✓		
36	38.8	327	MS^2^ [327]: 291 (29), 229 (32), 211 (22), 171 (100)	Oxo-dihydroxy-octadecenoic acid	✓	✓	✓	✓	✓	✓
37	40.6	329	MS^2^ [329]: 229 (100), 211 (88), 171 (97)	Trihydroxy-octadecenoic acid	✓	✓	✓	✓	✓	✓

* Identified by comparison with analytical standards. Hex = hexoside (usually glucoside, but also galactoside); Rut = rutinoside; dHex = deoxyhexoside (usually rhamnoside, but also furanoside).

**Table 3:** Quantification of the main compounds found in the extracts of *M. oleifera* food supplements analyzed.

Nº	Assigned Identification	mg g^−1^ DE					
		S1	S2	S3	S4	S5	S6
*Phenolic acids*							
5 + 6	Neochlorogenic + caffeic acid der.	0.76 ± 0.05 ^c^	0.42 ± 0.03 ^b^	0.29 ± 0.02 ^a^	0.43 ± 0.03 ^b^	0.87 ± 0.06 ^d^	0.23 ± 0.02 ^a^
9	3-*p*-Coumaroylquinic acid	0.18 ± 0.01 ^cd^	0.17 ± 0.01 ^bc^	0.21 ± 0.02 ^d^	0.27 ± 0.02 ^e^	0.112 ± 0.008 ^a^	0.14 ± 0.01 ^ab^
11 + 12	Chlorogenic acid + 3-FQA	0.78 ± 0.05 ^d^	0.34 ± 0.02 ^b^	0.38 ± 0.03 ^b^	0.54 ± 0.04 ^c^	0.76 ± 0.05 ^d^	0.18 ± 0.01 ^a^
**Total**		**1.72 ± 0.07 ^d^**	**0.93 ± 0.04 ^b^**	**0.88 ± 0.04 ^b^**	**1.24 ± 0.05 ^c^**	**1.74 ± 0.08 ^d^**	**0.55 ± 0.02 ^a^**
*Flavonoids*							
18	Vitexin	0.071 ± 0.005 ^b^	0.0096 ± 0.0007 ^a^	---	---	---	0.60 ± 0.04 ^c^
19 + 20	Rutin + isovitexin	0.64 ± 0.04 ^c^	1.00 ± 0.07 ^e^	0.034 ± 0.002 ^a^	0.49 ± 0.03 ^b^	---	0.85 ± 0.06 ^d^
21	Quercetin-*O*-Hex	4.0 ± 0.3 ^b^	1.40 ± 0.09 ^a^	1.8 ± 0.1 ^a^	4.7 ± 0.3 ^c^	5.2 ± 0.3 ^c^	1.7 ± 0.1 ^a^
22−25	Kaempferol + Quercetin glycosides	2.1 ± 0.1 ^c^	2.1 ± 0.1 ^c^	---	0.009 ± 0.001 ^a^	2.8 ± 0.2 ^d^	0.56 ± 0.04 ^b^
26	Kaempferol-*O*-Hex	1.9 ± 0.1 ^c^	2.4 ± 0.2 ^d^	0.56 ± 0.04 ^a^	1.12 ± 0.07 ^b^	2.5 ± 0.2 ^d^	0.60 ± 0.04 ^a^
27	Isorhamnetin-*O*-Hex	0.16 ± 0.01 ^a^	0.13 ± 0.01 ^a^	---	0.36 ± 0.02 ^b^	---	0.39 ± 0.03 ^b^
28	Quercetin-*O*-AHex	---	---	---	---	---	0.38 ± 0.02
29−31	Kaempferol + Quercetin glycosides	1.7 ± 0.1 ^b^	3.2 ± 0.2 ^d^	---	0.19 ± 0.01 ^a^	2.0 ± 0.1 ^c^	0.36 ± 0.02 ^a^
32 + 33	Kaempferol + Isorhamnetin-*O*-AHex	0.34 ± 0.03 ^c^	0.49 ± 0.03 ^d^	---	0.19 ± 0.01 ^a^	---	0.26 ± 0.02 ^b^
34	Kaempferol-*O*-AHex	---	---	---	---	---	0.25 ± 0.2
**Total**		**10.9 ± 0.4 ^d^**	**10.7 ± 0.3 ^d^**	**2.4 ± 0.1 ^a^**	**7.1 ± 0.3 ^c^**	**12.5 ± 0.4 ^e^**	**6.0 ± 0.3 ^b^**
**TIPC**		**12.6 ± 0.4 ^e^**	**11.7 ± 0.3 ^d^**	**3.3 ± 0.1 ^a^**	**8.3 ± 0.3 ^c^**	**14.2 ± 0.4 ^f^**	**6.6 ± 0.3 ^b^**

Values are reported as mean ± SD of three parallel experiments. Bold values represent the sum of each type of components. Means in the same line not sharing the same letter are significantly different at *p* < 0.05 probability level. Hex = hexoside (usually glucoside, but also galactoside); der. = derivative; FQA = feruloylquinic acid; AHex = acetylhexoside.

**Table 4:** Relative peak areas and heat map of extracts of *M. oleifera* food supplements.

Peak	Compound	S1	S2	S3	S4	S5	S6
1	Citric acid	0.00	0.00	3.65	1.16	0.00	0.00
2	Disaccharide	12.94	8.42	4.49	2.02	14.31	7.68
3	Glucomoringin	0.88	1.60	0.59	0.00	2.47	0.19
4	Hydroxytyrosol hexoside	0.09	0.14	0.17	0.00	0.22	0.30
5	Neochlorogenic acid	2.06	1.15	1.26	0.59	3.19	0.23
6	Caffeic acid derivative	0.54	0.22	0.29	0.17	0.29	0.11
7	Unknown	0.00	0.00	7.55	0.00	0.00	0.00
8	Unknown	0.00	0.00	0.00	0.00	0.00	0.00
9	3-*p*-Coumaroylquinic acid	0.35	0.40	1.56	0.50	0.31	0.38
10	Unknown	1.50	5.20	0.15	0.00	2.03	1.49
11	Chlorogenic acid	1.21	0.51	1.85	1.37	0.85	0.21
12	3-Feruloylquinic acid	0.21	0.21	0.32	0.11	0.37	0.19
13	Roseoside	1.10	0.05	0.00	0.06	0.11	0.28
14	4-*p*-Coumaroylquinic acid	0.17	0.10	2.40	0.91	0.12	0.18
15	Vicenin-2	1.23	1.33	3.92	0.87	1.33	3.41
16	Unknown	0.00	0.00	8.09	4.09	0.62	0.00
17	Unknown flavonoid-di-dHex	0.34	1.63	0.25	0.15	0.33	0.84
18	Vitexin 8-*C*-Glc-apigenin	1.61	0.73	0.96	0.75	0.83	1.95
19	Rutin	0.70	2.61	0.64	0.99	0.66	0.97
20	Isovitexin 6-*C*-Glc-apigenin	1.95	1.07	1.48	0.74	1.60	2.27
21	Quercetin-*O*-hexoside	20.58	10.31	26.16	38.12	17.82	27.54
22	Kaempferol-*O*-rutinoside	0.00	7.53	0.00	0.17	0.00	1.00
23	Quercetin-*O*-acetyl hexoside	10.38	3.90	0.30	0.24	10.09	3.91
24	Quercetin-malonyl hexose	1.58	0.75	0.00	0.00	1.99	0.91
25	Quercetin-der	0.31	0.47	0.25	0.30	0.28	0.00
26	Kaempferol-*O*-hexoside	17.40	19.52	17.40	18.83	17.91	13.80
27	Isorhamnetin-*O*-hexoside	0.96	0.65	3.39	2.76	1.28	1.41
28	Quercetin-*O*-acetyl hexoside	0.30	0.28	1.23	1.96	0.45	9.54
29	Kaempferol-der	0.30	1.96	0.17	0.27	0.00	3.93
30	Kaempferol-malonyl hexose	1.57	2.62	0	0.00	1.14	0.41
31	Kaempferol-*O*-acetyl hexoside	10.73	15.87	0.10	0.22	11.23	3.42
32	Kaempferol-*O*-acetyl hexoside	0.79	1.47	0.00	0.00	0.90	0.27
33	Isorhamnetin-*O*-acetyl hexoside	0.69	0.77	0.00	0.00	0.67	0.47
34	Kaempferol-*O*- acetyl hexoside	0.38	1.33	0.84	1.05	0.49	7.25
35	N-Feruloyltyramine	0.00	0.00	0.00	10.26	0.00	0.00
36	Oxo-dihydroxy-octadecenoic acid	5.31	5.48	4.78	5.25	4.28	4.64
37	Trihydroxy-octadecenoic acid	1.85	1.71	5.74	6.10	1.87	0.81

Hex = hexoside (usually glucoside, but also galactoside); Rut= rutinoside; dHex = deoxyhexoside (usually rhamnoside, but also furanoside); Glc = glucoside.

**Table 5:** Results (mmol TE/g DE) obtained in ABTS^·+^ and DPPH assays for *M. oleifera* food supplements. RSD (%) values in parenthesis.

Sample	ABTS^·+^	DPPH
S1	0.124 (20)	0.108 (16)
S2	0.128 (18)	0.104 (14)
S3	0.085 (19)	0.080 (14)
S4	0.105 (20)	0.108 (6)
S5	0.206 (5)	0.196 (8)
S6	0.069 (5)	0.052 (6)

**Table 6:** Results (IC50; mg DE/mL MeOH) obtained in ABTS^·+^ and DPPH assays. RSD (%) values in parenthesis.

Sample	ABTS^·+^	DPPH
S1	2.33 (16)	5.31 (14)
S2	2.11 (15)	5.59 (12)
S3	3.08 (14)	7.14 (12)
S4	2.58 (20)	5.22 (6)
S5	1.26 (5)	2.77 (7)
S6	3.76 (5)	10.47 (6)

## Data Availability

The data presented in this study are available in the article and Supplementary Materials.

## Supplementary Materials

The following supporting information can be downloaded at: https://www.mdpi.com/article/10.3390/antiox12010110/s1, Figure S1: HPLC-ESI-MS base peak chromatogram of the analytical standards neochlorogenic acid (1), chlorogenic acid (2), vicenin-2 (3), coumaric acid (4), rutin (5), quercetin (6) and kaempferol (7).; Table S1: Results (mg TE/100g DE) obtained in ABTS·+ and DPPH assays. RSD (%) values in parenthesis.
