# Supplementary material for: Preliminary Phytochemical Screening and Antioxidant Activity of Commercial Moringa oleifera Food Supplements
Source: Antioxidants (Basel). 2023 Jan 2;12(1):110. doi: 10.3390/antiox12010110 (PMC9855063; doi:10.3390/antiox12010110)
Supplement: Supplementary file 1 [file antioxidants-12-00110-s001.zip › antioxidants-2067023-supplementary.pdf]

Supplementary Material

Phytochemical profile and antioxidant activity of *Moringa Oleifera* commercial food supplements. A source of antioxidants in your diet

Eulogio J. Llorent-Martínez*, Ana I. Gordo-Moreno, M.L. Fernández-de Córdova, Antonio Ruiz-Medina

Department of Physical and Analytical Chemistry, Faculty of Experimental Sciences, University of Jaén, Campus Las Lagunillas, E-23071 Jaén, Spain

*Correspondence: ellorent@ujaen.es; Tel.: +34-953-212-938

1. Chromatographic analysis

The HPLC system was an Agilent Series 1100, composed of a vacuum degasser, an autosampler, a binary pump, and a G1315B diode array detector (Agilent Technologies, Santa Clara, CA, USA). We used a reversed phase Luna Omega Polar C_{18} analytical column of 150 x 3.0 mm and 5 µm particle size (Phenomenex, Torrance, CA, USA) and a Polar C_{18} Security Guard cartridge (Phenomenex) of 4 x 3.0 mm. The mobile phases consisted of water + formic acid 0.1 % v/v (eluent A) and acetonitrile (eluent B). The gradient elution was: 10-25% B in 0-25 min, 25% B in 25-30 min, 25-50% B in 30-40 min, 50-100% B in 40-42 min, 100% in 42-47 min. Then, eluent B was returned to 10% with a 7 min stabilization time. The flow rate was 0.4 ml min^{-1}.

The HPLC system was connected to an ion trap mass spectrometer (Esquire 6000, Bruker Daltonics, Billerica, MA, USA) equipped with an electrospray ionization interface. The scan range was at *m/z* 100–1200 with a speed of 13,000 Da/s. The ESI conditions were: drying gas (N_2) flow rate and temperature, 10 L/min and 365 ºC; nebulizer gas (N_2) pressure, 50 psi; capillary voltage, 4500 V; capillary exit voltage, -117.3 V. We used the auto MS^n mode for the acquisition of MS^n data, with isolation width of 4.0 *m/z,* and fragmentation amplitude of 0.6 V (MS^n up to MS^4).

2. Chemicals and reagents

All reagents and standards were of analytical reagent grade. Standards of chlorogenic acid, neochlorogenic acid, coumaric acid, quercetin, kaempferol, rutin, and vicenin-2 were purchased from Merck (Madrid, Spain). All solutions were prepared in methanol (MeOH) HPLC-grade (Sigma-Aldrich). LC-MS grade acetonitrile (Panreac; Barcelona, Spain) and ultrapure water (Milli-Q Waters purification system; Millipore; Milford, MA, USA) were also used. A chromatogram of the analytical standards used is shown in Figure S1.

Ethanol (96%), 2,2′-azinobis(3-ethylbenzthiazoline-6-sulfonic acid) (ABTS; ≥98%), 2,2-diphenyl-1-picrylhydrazyl (DPPH; 95%), gallic acid monohydrate (>98%) and potassium persulfate (>99%) were obtained from Merck.

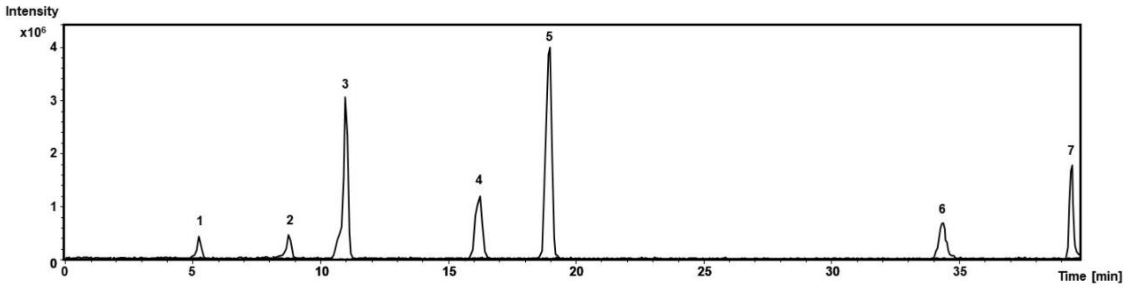

Figure S1. HPLC-ESI-MS base peak chromatogram of the analytical standards neochlorogenic acid (1), chlorogenic acid (2), vicenin-2 (3), coumaric acid (4), rutin (5), quercetin (6) and kaempferol (7).

3. Determination of antioxidant capacity

In $ABTS^{\cdot+}$ assay, the $ABTS^{\cdot+}$ solution was prepared by reacting 50 mL of 2 mM $ABTS^{\cdot+}$ solution with 200 µL of 70 mM potassium persulfate solution. After 16 h in the dark at room temperature, the $ABTS^{\cdot+}$ solution was diluted with ethanol to an initial absorbance of 0.70 ± 0.02 measured at 734 nm. Sample solution (100 µL) was mixed with 1.8 mL of $ABTS^{\cdot+}$ solution. After 10 min in the dark, the absorbance was measured at 734 nm.

In DPPH assay, 100 µL of the sample solution was added to 3.5 mL of a 0.06 mM MeOH DPPH radical solution. After 30 min in the dark, the absorbance was measured at 516 nm.

Table S1. Results (mg TE/100g DE) obtained in $ABTS^{\cdot+}$ and DPPH assays. RSD (%) values in parenthesis.

Sample	$ABTS^{\cdot+}$	DPPH
S1	3.1 (20)	2.7 (16)
S2	3.2 (18)	2.6 (14)
S3	2.1 (19)	2 (14)
S4	2.6 (20)	2.7 (6)
S5	5.2 (5	4.9 (8)
S6	1.7 (5)	1.3 (6)
